# Authors’ Reply

**DOI:** 10.1055/s-0039-1683361

**Published:** 2019-03

**Authors:** Maria Lúcia Moleiro, Luís Guedes-Martins, Alexandrina Mendes, Cláudia Marques, Jorge Braga

**Affiliations:** 1 Woman’s and Reprodutive Medicine Department, Centro Materno Infantil do Norte, Centro Hospitalar Universitário do Porto, Porto, Portugal

Comments on: Modified Pereira Suture as an Effective Option to Treat Postpartum Hemorrhage Due to Uterine Atony

Dear Editor,

The modification of the Pereira suture described by our team was performed during a cesarean section, when uterine atony was noted and was not responsive either to uterotonic drugs or to bimanual uterine massage. Given the success rates of uterine compression sutures (UCSs) described and the possibility of uterus preservation, they were the chosen method.

Since hysterotomy had already been performed, the B-Lynch suture was the first technique used.^1^ As the uterine atony persisted, the Pereira technique was chosen due to its characteristics: an even distribution of pressure around the uterus, due to the small bites at regular intervals in the uterine wall; not entering the uterine cavity, thus reducing the risk (at least theoretically) of uterine synechiae and infection; the ability to prevent the sliding out of the thread after uterus involution, reducing the risk of bowel and intestine entrapment.^2^

The emergent need for hemorrhage control in cases of uterine atony limits the existence of quality evidence stating superiority amongst UCS techniques. Nevertheless, previous reviews report success rates > 75% (some of them > 90%), regardless of technique.^3^ ^4^ ^5^ ^6^ In these works, variations of the B-Lynch suture were reported as being as effective or even superior to the original technique. However, the chosen technique may not be the only variable contributing to the disparity of results, and the timing in which uterine compression sutures are applied, as well as surgeons’ experience, is of vital importance.^3^ ^6^ ^7^ ^8^ ^9^ ^10^ ^11^ ^12^

The two cases reported by our team do not intend to describe a new technique, but rather to support UCSs as an adequate option to treat postpartum hemorrhage due to uterine atony, always bearing in mind the characteristics of each situation and the specificities of each suture.
